# Urban-rural differences in the prevalence of non-communicable diseases risk factors among 25–74 years old citizens in Yangon Region, Myanmar: a cross sectional study

**DOI:** 10.1186/s12889-016-3882-3

**Published:** 2016-12-05

**Authors:** Aung Soe Htet, Marius B. Bjertness, Lhamo Y. Sherpa, Marte Karoline Kjøllesdal, Win Myint Oo, Haakon E. Meyer, Hein Stigum, Espen Bjertness

**Affiliations:** 1 Department of Community Medicine and Global Health, Institute of Health and Society, Faculty of Medicine, University of Oslo, Oslo, Norway; 2 International Relations Division, Ministry of Health, Nay Pyi Taw, Myanmar; 3 Faculty of Medicine, SEGi University, Petaling Jaya, Malaysia

**Keywords:** Risk factors, Non-communicable diseases, Urban-rural differences, Yangon, Myanmar

## Abstract

**Background:**

Recent societal and political reforms in Myanmar may upturn the socio-economy and, thus, contribute to the country’s health transition. Baseline data on urban-rural disparities in non-communicable disease (NCD) risk factors are not thoroughly described in this country which has been relatively closed for more than five decades. We aim to investigate urban-rural differences in mean values and the prevalence of selected behavioral and metabolic risk factors for non-communicable diseases and 10-years risk in development of coronary heart diseases (CHD).

**Methods:**

Two cross-sectional studies were conducted in urban and rural areas of Yangon Region in 2013 and 2014 respectively, using the WHO STEPwise approach to surveillance of risk factors of NCDs. Through a multi-stage cluster sampling method, 1486 participants were recruited.

**Results:**

Age-standardized prevalence of the behavioral risk factors tended to be higher in the rural than urban areas for all included factors and significantly higher for alcohol drinking (19.9% vs. 13.9%; *p* = 0.040) and low fruit & vegetable consumption (96.7% vs. 85.1%; *p* = 0.001). For the metabolic risk factors, the tendency was opposite, with higher age-standardized prevalence estimates in urban than rural areas, significantly for overweight and obesity combined (40.9% vs. 31.2%; *p* = 0.023), obesity (12.3% vs.7.7%; *p* = 0.019) and diabetes (17.2% vs. 9.2%; *p* = 0.024). In sub-group analysis by gender, the prevalence of hypercholesterolemia and hypertriglyceridemia were significantly higher in urban than rural areas among males, 61.8% vs. 40.4%; *p* = 0.002 and 31.4% vs. 20.7%; *p* = 0.009, respectively. Mean values of age-standardized metabolic parameters showed higher values in urban than rural areas for both male and female. Based on WHO age-standardized Framingham risk scores, 33.0% (95% CI = 31.7–34.4) of urban dwellers and 27.0% (95% CI = 23.5–30.8) of rural dwellers had a moderate to high risk of developing CHD in the next 10 years.

**Conclusion:**

The metabolic risk factors, as well as a moderate or high ten-year risk of CHD were more common among urban residents whereas behavioral risk factors levels were higher in among the rural people of Yangon Region. The high prevalences of NCD risk factors in both urban and rural areas call for preventive measures to reduce the future risk of NCDs in Myanmar.

## Background

Non-communicable diseases (NCDs) such as cardiovascular diseases, cancer, chronic lung diseases, and diabetes, are the primary causes of morbidity and mortality both globally and in the South-East Asia region (SEAR) [1, 2].

In 2012, NCDs were responsible for 38 million deaths (68%) globally [1] and the figure is expected to increase to 52 million deaths by 2030 [1, 3]. More than 80% of NCD deaths occurred within low and middle income countries, and approximately 42% of deaths occurred before the age of 70 years [1]. In SEAR, NCDs are now causing 7.9 million deaths annually, or 55% of total deaths [2]. Globally [4], and in SEAR [5], an increased burden of NCD risk factors has been observed during the two decades from 1990 to 2010 [4], concomitant with a shift in disease pattern from infectious diseases to chronic diseases due to a more westernized lifestyle and urbanization. In Myanmar 59% of total deaths was due to NCDs [6].

Urbanization is expected to increase in the developing countries including Asian countries [7], reducing the number of people living in traditional rural livelihoods. Based on the Myanmar census 2014, 70% of the population resides in the rural areas [8]. Currently the annual urbanization rate in Myanmar is 1.6% [7]. Furthermore, the recent societal and political changes in Myanmar [9] may upturn the economy, change social structures and alter lifestyles practices. Socioeconomic improvements are important drivers of a health transition [10, 11] leading to changes in disease pattern and demography. These changes will have implication for the emergence of NCD and their risk factors [12]. The global leading NCD risk factors ranked according to their contribution to global death, are high blood pressure, tobacco use, high blood glucose, low physical activity, overweight and obesity, high blood cholesterol and alcohol use [13]. Based on a national STEPwise approach to NCD risk factor surveillance (STEPs) survey in Myanmar in 2009, the prevalence of daily smokers, current alcohol drinkers, low fruit and vegetable intake, low physical activity, overweight and obesity, and hypertension were 17, 13, 90, 13, 25 and 30%, respectively [14, 15].

In the Myanmar National Health Plan (2011–2016) [16], tackling NCDs was set as a priority, and separate programs on the different NCDs, including cardiovascular diseases, diabetes and cancer, have been put in place. However, an operational integrated NCD strategy recommended by World Health Organization (WHO) has not been developed [5]. A greater understanding of NCD determinants including urban-rural differences is essential in the NCD prevention mechanism to prioritize actions and to tailor strategies according to the available resources. Solid data on NCD risk factors can be utilized for estimation of development of chronic diseases. Several studies including from Asia countries [17–20] predicted the development of coronary heart disease (CHD) based on estimates of major risk factors of NCD. Published papers, including NCD risk factors and urban-rural disparities in Myanmar, are scarce due to constraints in research and international publications during the administration under military from 1962 [21] to 2011.

Using two cross-sectional studies of 25–74 years old male and female from urban and rural Yangon region from 2013 and 2014, the present paper aims at investigating urban-rural differences in the prevalence of selected risk factors for NCDs, such as smoking, alcohol consumption, low fruit and vegetable intake, low physical activity, overweight, obesity, hypertension, diabetes and abnormal lipid profiles. We also aim at comparing mean level of metabolic parameters between urban and rural areas. We hypothesize that the prevalence of NCD risk factors and metabolic parameters are higher among the urban than the rural population. Furthermore, we aim at estimating 10 years risk for development of CHD based on the Framingham risk score in urban and rural areas.

## Methods

Two household based cross-sectional studies were conducted in urban and rural areas of Yangon region between September and November of 2013 and 2014, respectively. The Yangon region was purposely chosen from the 14 states and regions of Myanmar. Yangon region is located in the lower part of Myanmar with a population of 7.4 million, which constitutes 14.3% of the national population [8]. In Yangon it is estimated that 5.2 million lives in urban and 2.2 million in rural area [8]. The region is made up of 27 urban townships and 18 rural townships.

The methodology of the present study is done in accordance with the WHO STEPs wise approach for the surveillance of chronic disease risk factors [22]. We included all three STEPs: STEP (1) Questionnaire survey including socio-demographic characteristics, smoking, alcohol consumption, physical activity, fruit and vegetable consumption, history of hypertension and diabetes; STEP (2) Physical measurement including blood pressure, body height, weight, waist and hip circumference; STEP (3) Laboratory investigation of venous blood samples for fasting lipid profiles, including total cholesterol (TC), triglycerides (TG), high-density lipoprotein (HDL), and fasting blood glucose.

### Sampling

Men and women aged 25–74 years were included in the study. Exclusion criteria were being physically and mentally too ill to participate. Institutionalized people, military personals, Buddhist monks and nuns were not included in the study. Based on WHO sample size calculator for STEP survey [22], it is estimated that in each study we need a sample size of 500 to estimate the prevalence of most of the risk factors (e.g., prevalence around 10% for hypertension, physical inactivity and around 90% for low fruit and vegetable consumption), while other risk factors like smoking in males and overweight with prevalence between 20–25% require a sample size of 1000 with a level of confidence of 1.96, margin of error of 0.05, the design effect 1.5 and an expected response rate of 80% [14]. Based on these numbers and practical limitations, we estimated that a sample size of 800 in each of the two studies would give fairly precise estimates, covering most risk factors. Sample sizes were estimated for the urban and rural survey separately, and for the purpose of sub-group analysis by gender.

A multi-stage cluster sampling method was performed. Six townships from urban areas and six townships from rural areas were randomly selected. Each township is built up with villages in rural areas and wards (urban township unit) in urban areas. From each selected township, five wards or villages were randomly selected (totally 30 wards and 30 villages). From each selected ward and village, 26–27 households were randomly chosen, and from the households, the eligible members were listed, and one was randomly invited to participate in the study. From half of the selected household male participants were selected and from the other half, female participants. Data was collected during the daytime of the first visit, and then in the morning of the next day for blood drawing. If a selected participant was not at home, s/he was contacted and an appointment was made for the next day. In such cases, the interviewing and physical measurements were most often performed right after the blood samples were taken. A total of 1608 persons with equal sex distribution (804 from urban and 804 from rural) were invited. Out of them, a total of 755 (94%) from urban areas and of 731 (91%) from rural areas completed STEP (1) and (2). A total of 692 (86%) urban participants and 676 (83%) rural participants finished all three STEPs. Most common reasons for not participating in STEP 1 and 2 were “not willing” and “not having time”. In STEP 3, non-response was due to worries about blood test. We excluded 13 pregnant women (3 from urban and 10 from rural) because maternal physiological changes due to pregnancy might affect estimates.

## Data collection and measurements

Five trained medical doctors collected data in the urban study and in the rural study. The questionnaire was previously translated into Myanmar language from the WHO STEPs Instrument version 2.1 [22]. A two-days training and pre-test were conducted in both urban and rural studies. Standardization of the instruments used in the field work was done before and during the training. The field workers conducted a pilot of STEP 1 and STEP 2, in 20 male and female aged 25–74 years from different level of socio-economic and educational status in urban and rural areas. Questions and comments were clarified during and at end of the pilot. After the clarification, the field workers performed a trial in practical skills. Show cards were developed and used to identify and explain the questions in tobacco products, alcohol consumption, diet and physical activities [22]. We used identical methods for training and for practice in both surveys following the STEPs methodology [22]. Participants were interviewed about socio-demography and behavioral risk factors, followed by blood pressure and anthropometric measurements. Blood pressure was measured three times with 3 min pause, 15 min after face-to-face interview, using OMRON M6 automatic blood pressure monitor. Measuring tape (SPEED-5 M) was used to measure individual’s body height without foot wear and any head gear, to the nearest to 0.1 cm (cm) [22]. Body weight was measured with a portable Equinox BR-9808 weighing scale and noted in kilogram (kg) to the nearest 0.1 kg. The participants were requested to wear light clothes without footwear during weighing. Waist and hip circumference measurement were conducted in a private place with the soft measuring tape. Waist circumference was measured in centimeter in the standing position over the skin directly at midpoint between the lower edge of the last palpable rib and the top of the hip bone [22]. The hip circumference was taken in centimeter at maximum circumference over buttocks horizontally [22]. Measures were done to the nearest 0.1 cm.

The participants were requested a minimum 10 h overnight fasting before participants the next morning met for blood drawing in a nearby health facility or survey site. Venous blood samples were collected in lipid tube and glucose tube, that contained fluoride and carried in cold boxes with ice and transported to the National Health Laboratory, Yangon, a reference laboratory of Ministry of Health. Biochemical analysis was conducted less than three hours after blood samples were taken.

Fasting blood glucose level was measured by the enzymatic reference method with hexokinase, using reagents of COBAS from Roche Diagnostics, Indianapolis, IN. Fasting blood concentrations of total cholesterol, triglycerides and HDL were determined by the enzymatic colorimetric test, using reagents of COBAS from Roche Diagnostics, Indianapolis, IN.

## Variables

### Socio-demographic factors

Based on the ward or village tract administration Law 2012 [23], we define a ward as an urban unit and a village as a rural unit. In this study, urban area was confined within urban townships that have no rural unit, constituting the Yangon city, the largest and the former capital of Myanmar. Rural area was defined as the villages belonging to rural townships outside the Yangon city. Residents in the wards of rural townships were not included, as they were considered as semi-urban.

*Age* was defined as the completed year of age.

In the questionnaire, we asked for both years at school and highest education level. Highest *education level* includes seven categories; no formal school, less than completed primary school, primary school completed (5 years), secondary school completed (9 years), high school completed (11 years), college/university (14 years and above) and post-graduate degree (15 years and above).

Daily personal *income* was calculated from household income divided by number of household members and converted into United State Dollar (USD) from local currency Kyats. We used the World Bank’s cut-off-value of poverty lines 1.90 USD/day and 3.10 USD/day [24, 25].

### Behavioural risk factors

*Daily smoker* was defined as those who currently smoked tobacco daily. *Daily smokeless tobacco user* was defined as those who currently used any smokeless tobacco such as snuff, chewing tobacco and betel quid on a daily basis. *Current alcohol drinker* was defined as those who have consumed alcohol at least once in the previous 30 days. *Heavy alcohol drinker* was defined as who had average pure alcohol consumption more than 40 g/day for male and more than 20 g alcohol/day for female [22]. Show-cards were used for identifying tobacco products and an estimation of the amount of alcohol consumption.

*Fruit and vegetable consumption*: In accordance with WHO recommendation, low fruit and vegetable intake was defined as those who had less than a combination of five servings of fruit and vegetable on average per day [22]. One standard serving was equivalent to approximately 80 g of fruit or vegetable [22]. Show-cards were used for estimation of the amount of servings.

Physical activity was estimated across all domains of work, household tasks, transportation and leisure-time activity. *Low physical activity* was defined as less than 3 days of vigorous-intensity activity of at least 20 min per week, or less than 5 days of moderate- intensity activity (with a minimum of at least 600 MET-minutes) per week, using standard metabolic equivalent of tasks (MET) based on WHO guidelines [22]. Types of physical activities were identified using the typical examples from show-cards.

### Metabolic risk factors

Body Mass Index (BMI) was calculated as weight in kilograms divided by the height in meters squared. *Overweight* was defined as BMI of 25–29.9 kg/m^2^ and obesity was defined as a BMI of ≥ 30 kg/m^2^ [22]. *Central obesity* was defined as a waist-hip ratio above 0.90 for males and above 0.85 for females [26]. *Hypertension* was defined as having an average of systolic blood pressure ≥140 mmHg and/or diastolic blood pressure ≥ 90 mmHg, and/or self-reported current anti-hypertensive treatment for hypertension within 2 weeks prior to the interview [22]. Mean blood pressure was calculated from the average of the second and third measurements.

*Diabetes* was defined as a fasting blood glucose level of ≥ 7 mmol/L and/or self-reported diabetes [27]. Self-reported diabetes included only those diagnosed by a medical doctor or other health worker.

The cut-off for *hypercholesterolemia* was total cholesterol level ≥5.17 mmol/L according to the abnormal lipid profile criteria reported by National Cholesterol Education Programme (NCEP) [28].

*Hypertriglyceridemia* was defined as triglyceride level ≥2.0 mmol/L [22].

*The Framingham risk score* was computed using a gender-specific algorithm to estimate the 10-year risk of developing coronary heart diseases [17, 18]. The risk score included age, total cholesterol, high-density lipoprotein, systolic and diastolic blood pressure, diabetes and smoking [17, 18]. The score is previously validated in a multiethnic Asian population [19, 20].

## Statistical methods

Double data entry was performed using Epidata software version 3.1. STATA/IC version 14.0 was used in analysis of data. We declared the complex sample design using “svyset” command in STATA identifying the sample design with specified weighing of different stages of sampling units based on the study population according to the 2014 Myanmar census data [8]. Following the prefix “svy” command, mean and proportions were computed with age-standardization of the study population incorporating complex survey design. Age-standardized prevalence was calculated for the major risk factors by the direct standardization method based on the study population (internal standardization) and the WHO world standard population (external standardization) [29]. Statistical differences in age-standardized mean of metabolic measurements and age-standardized proportions of NCD risk factors between urban-rural locations were tested by the Wald test.

## Results

The urban population was more educated, and two years older than their rural counterparts (Table 1). The proportion with income less than 1.9 USD per day was high in both urban (46%) and rural areas (67%).

**Table 1:** Socio-demographic factors of 25–74 years old citizens in Yangon region, by urban-rural location

	Urban (*n* = 755)	Rural (*n* = 731)	Total (*n* = 1486)	*p*-value^#^
Gender (%)				
Male	376 (49.8)	369 (50.5)	745 (50.1)	
Female	379 (50.2)	362 (49.5)	741 (49.9)	
Age (Mean Years ± SD)				
Male	49.9 ± 13.9	46.8 ± 12.5	48.4 ± 13.3	0.001 *
Female	47.5 ± 12.5	46.2 ± 12.4	46.9 ± 12.4	0.131
Total	48.7 ± 13.2	46.5 ± 12.4	47.6 ± 12.9	0.001 *
Age group (%)				
25–34	142 (18.8)	144 (19.7)	286 (19.2)	0.526
35–44	160 (21.2)	190 (26.0)	350 (23.6)	
45–54	171 (22.6)	180 (24.6)	351 (23.6)	
55–64	178 (23.6)	151 (20.7)	329 (22.1)	
65–74	104 (13.8)	66 (9.0)	170 (11.4)	
Education (Mean Years ± SD)				
Male	10.8 ± 3.8	6.1 ± 3.9	8.5 ± 4.5	<0.001 *
Female	9.7 ± 4.4	5.4 ± 3.7	7.6 ± 4.6	<0.001 *
Total	10.2 ± 4.1	5.7 ± 3.8	8.0 ± 4.6	<0.001 *
Highest Education Level (%)				
No formal school	20 (2.6)	70 (9.6)	90 (6.1)	
Less than Primary school	35 (4.6)	188 (25.7)	223 (15.0)	
Primary school completed	167 (22.1)	301 (41.2)	468 (31.5)	
Secondary school completed	176 (23.3)	102 (14.0)	278 (18.7)	
High school completed	157 (20.8)	29 (4.0)	186 (12.5)	
College/university completed	185 (24.5)	26 (3.6)	211 (14.2)	
Post graduate degree	15 (2.0)	13 (1.8)	28 (1.9)	<0.001 *
Income (%) ^a^				
Less than 1.9 USD per day	313 (45.5)	470 (66.5)	783 (56.1)	
1.9 to <3.1 USD per day	156 (22.7)	126 (17.8)	282 (20.2)	
3.1 USD per day and above	219 (31.8)	111 (15.7)	330 (23.7)	<0.001 *

^#^ *p*- value for differences between urban and rural values *p-value <0.05 from Wald test

^a^Missing respondents - 91 due to refuse; (exchange rate:1 USD = 953.8 Myanmar Kyat as of 05-11-2013)

The prevalence of behavioral NCD risk factors (daily smoker, smokeless user, current alcohol drinker, low fruit & vegetable intake and low physical activity) were generally lower in urban than rural areas while the metabolic risk factors (overweight, obesity, hypertension, diabetes, hypercholesterolemia and hypertriglyceridemia) were higher in urban than rural areas (Table 2). Except for low physical activity, age-standardized prevalence estimates adjusted to the WHO-population showed similar trends (Table 2). In sub-group analysis by gender (Table 3), the urban-rural differences in risk factors further demonstrated the pattern of higher prevalence of metabolic risk factors in urban areas and the higher prevalence of behavioral risk factors in rural areas.

**Table 2:** Internal age-standardized and WHO-age-standardized prevalence of behavioral^a^ and metabolic risk factors^b^ for NCDs among 25–74 years old citizens in Yangon region, by urban-rural location

	Age-standardized prevalence			Age-standardized prevalence to WHO population	
	Urban	Rural	*p*-value^#^	Urban	Rural
	% (95% CI)	% (95% CI)		% (95% CI)	% (95% CI)
Daily smoker^a^	21.5 (13.0–30.1)	27.0 (23.0–31.1)	0.227	21.1 (15.1–28.6)	26.0 (22.1–30.4)
Daily smokeless user^a^	28.0 (21.6–34.3)	32.4 (29.2–35.5)	0.198	29.2 (22.2–37.5)	32.7 (29.4–36.1)
Current alcohol drinker^a^	13.9 (11.9–16.0)	19.9 (14.6–25.1)	0.040 *	16.2 (13.9–18.8)	21.2 (15.8–27.9)
Heavy drinker^a^	3.5 (2.5–4.4)	4.8 (1.6–7.9)	0.394	4.0 (3.0–5.3)	5.5 (2.5–11.7)
Low fruit & vegetable intake^a^	85.1 (79.6–90.6)	96.7 (95.3–98.1)	0.001 *	85.1 (78.9–89.7)	96.6 (95.0–97.6)
Low physical activity^a^	10.7 (6.4–15.0)	11.1 (7.6–14.6)	0.873	9.7 (5.9–15.5)	9.2 (6.6–12.7)
Overweight and obesity^b^	40.9 (33.5–48.2)	31.2 (27.9–34.5)	0.023 *	38.9 (31.1–47.4)	30.1 (26.2–34.3)
Obesity^b^	12.3 (8.9–15.7)	7.7 (6.2–9.2)	0.019 *	11.5 (9.2–14.4)	6.9 (5.3–8.9)
Central obesity^b^	33.1 (25.2–41.0)	37.0 (31.2–42.7)	0.396	29.2 (22.2–37.3)	34.3 (29.2–39.9)
Hypertension^b^	41.1 (37.6–44.6)	39.7 (33.0–46.5)	0.697	37.4 (34.2–40.8)	35.3 (29.8–41.3)
Diabetes^b^	17.2 (10.9–23.5)	9.2 (7.0–11.5)	0.024 *	14.1 (9.0–21.3)	7.2 (5.8–8.9)
Hypercholesterolemia^b^	56.0 (49.2–62.8)	47.3 (40.5–54.2)	0.074	52.7 (45.6–59.7)	43.2 (35.4–51.3)
Hypertriglyceridemia^b^	22.8 (19.3–26.4)	19.1 (10.6–27.7)	0.394	20.6 (16.8–24.9)	17.9 (10.5–28.9)

Daily smoker: currently smoke tobacco daily, Daily smokeless user: who currently daily-used any smokeless tobacco such as snuff, chewing tobacco and betel quid, current alcohol drinker- current drinker within 30 days, Heavy alcohol drinker: average alcohol intake > 40 g/day for male and >20 g/day for female, Low fruit & vegetable intake: < 5 servings of fruits and/or vegetables on an average per day, Low physical activity: less than 3 days of vigorous-intensity activity of at least 20 min per week, or less than 5 days of moderate- intensity activity (with a minimum of at least 600 MET-minutes) per week, overweight: BMI 25–29.9 kg/m^2^, obesity: BMI ≥30 kg/m^2^), Central obesity: waist-hip ratio >0.90 for male and > 0.85 for female, Hypertension: systolic blood pressure ≥140 and/or diastolic blood pressure ≥90 mmHg or currently on medication for raised BP, Diabetes: Raised fasting blood glucose ≥ 7.0 mmol/L and/or history of diabetes, Hypercholesterolemia: ≥ 5.17 mmol/L of total cholesterol, Hypertriglyceridemia: ≥ 2.0 mmol/L of triglycerides

^#^ *p*- value for differences between urban and rural values *p-value <0.05 from Wald test

^a^Behavioral risk factor, ^b^metabolic risk factor

**Table 3:** Internal age-standardized prevalence of behavioral^a^ and metabolic risk factors^b^ for NCDs among 25–74 years old citizens in Yangon region, by sex and urban-rural location

	Male			Female		
	Urban	Rural	*p*-value^#^	Urban	Rural	*p*-value^#^
	% (95% CI)	% (95% CI)		% (95% CI)	% (95% CI)	
Daily smoker^a^	36.2 (28.4–44.2)	42.8 (35.7–49.8)	0.201	5.5 (1.7–12.7)	12.9 (10.6–15.1)	0.003*
Daily smokeless user^a^	39.4 (32.8–46.1)	46.9 (45.3–48.6)	0.034*	16.4 (10.2–22.7)	18.3 (11.9–24.7)	0.305
Current alcohol drinker^a^	28.4 (26.3–30.5)	39.0 (30.8–47.1)	0.018*	1.0 (0.2–2.3)	0.3 (0.0–0.8)	0.254
Heavy drinker^a^	6.5 (5.1–7.9)	9.8 (5.0–14.7)	0.178	0	0	0
Low fruit &vegetable intake^a^	83.8 (77.1–90.5)	97.8 (95.5–99.9)	0.001*	86.5 (82.9–90.2)	95.8 (93.6–98.0)	0.006*
Low physical activity^a^	12.3 (4.3–20.2)	9.7 (4.1–15.3)	0.572	9.1 (4.4–13.8)	12.7 (9.7–15.7)	0.387
Overweight and obesity^b^	33.4 (24.4–42.4)	19.4 (16.1–22.7)	0.008*	48.9 (41.9–56.0)	43.8 (41.9–56.0)	0.038*
Obesity^b^	6.9 (3.8–10.0)	3.8 (2.8–4.9)	0.061	17.3 (14.1–20.5)	11.5 (9.8–13.2)	<0.001*
Central obesity^b^	33.1 (20.8–45.4)	32.6 (28.0–37.2)	0.941	33.4 (24.1–42.7)	41.4 (33.4–49.4)	0.235
Hypertension^b^	43.1 (39.3–47.0)	37.9 (30.5–45.3)	0.190	42.2 (35.6–48.9)	41.5 (32.8–50.2)	0.587
Diabetes^b^	16.7 (10.4–23.1)	7.4 (4.9–10.0)	0.012*	17.7 (9.1–26.4)	10.8 (7.1–14.6)	0.049*
Hypercholesterolemia^b^	61.8 (51.9–71.7)	40.4 (34.0–46.9)	0.002*	50.7 (42.6–58.9)	54.2 (42.7–65.7)	0.506
Hypertriglyceridemia^b^	31.4 (26.7–36.0)	20.7 (14.8–26.5)	0.009*	18.4 (13.8–23.0)	18.9 (8.5–29.3)	0.795

Daily smoker: currently smoke tobacco daily, Daily smokeless user: who currently daily-used any smokeless tobacco such as snuff, chewing tobacco and betel quid, current alcohol drinker- current drinker within 30 days, Heavy alcohol drinker: average alcohol intake > 40 g/day for male and >20 g/day for female, Low fruit & vegetable intake: < 5 servings of fruits and/or vegetables on an average per day, Low physical activity: less than 3 days of vigorous-intensity activity of at least 20 min per week, or less than 5 days of moderate- intensity activity (with a minimum of at least 600 MET-minutes) per week, overweight: BMI 25–29.9 kg/m^2^, obesity: BMI ≥30 kg/m^2^), Central obesity: waist-hip ratio >0.90 for male and > 0.85 for female, Hypertension: systolic blood pressure ≥140 and/or diastolic blood pressure ≥90 mmHg or currently on medication for raised BP, Diabetes: Raised fasting blood glucose ≥ 7.0 mmol/L and/or history of diabetes, Hypercholesterolemia: ≥ 5.17 mmol/L of total cholesterol, Hypertriglyceridemia: ≥ 2.0 mmol/L of triglycerides

^#^ *p*- value for differences between urban and rural values, *p-value <0.05 from Wald test

^a^Behavioral risk factor, ^b^metabolic risk factor

The prevalence of alcohol drinking and low fruit and vegetable intake were significantly lower in urban than in rural area, 13.9% vs. 19.9% and 85.1% vs. 96.7%, respectively (Table 2). Overall, we found no significant difference between urban and rural residents in the prevalence of physical inactivity (Table 2). Both in urban and rural areas, most of the physical activity was related to work and little to leisure time. However, vigorous activity in workplace was lower among urban residents than among the rural residents, while for leisure time physical activity it was opposite: higher in urban than in rural residents (not shown in table). Regarding daily smoking and use of smokeless tobacco, there was no significant difference in prevalence between urban and rural areas (Table 2). However, in sub-group analysis by urban-rural location and sex, the daily smoking prevalence was found to be significantly lower in urban women (5.5%) than in rural women (12.9%) (Table 3). Similarly, among males the prevalence of daily smokeless tobacco user was lower in urban than rural areas 39.4% vs. 46.9%. The majority of urban smokers (60%) smoked 2–10 cigarettes daily while 94% of rural smokers used 1–10 local made cheroots daily (data not shown in Tables).

The overall prevalences of metabolic risk factors were significantly higher in urban than in rural areas for overweigh and obesity combined (40.9% vs. 31.2%), obesity (12.3% vs. 7.7%), and diabetes (17.2% vs 9.2%). Regarding sub-group analysis of the metabolic risk factors, urban women had significantly higher prevalence than rural women for overweight and obesity combined (48.9% vs 43.8%), obesity (17.3% 11.5%) and diabetes (17.7% vs. 10.8%) (Table 3). For males, urban prevalences were significantly higher than rural for overweight and obesity combined (33.4% vs.19.4%), diabetes (16.7% vs. 7.4%), hypercholesterolemia (61.8% vs. 40.4%) and hypertriglyceridemia (31.4% vs. 20.7%).

The age-standardized mean values of BMI, fasting blood glucose, blood cholesterol, triglyceride and high-density lipoprotein showed significantly more unfavourable values in the urban than the rural populations (Table 4). No significant difference was reported in blood pressure.

**Table 4:** Internal age-standardized mean values of metabolic risk factors for NCDs among 25–74 years old citizens in Yangon region, by sex and urban-rural location

	Male			Female			Total		
	Urban	Rural	*p*-value^#^	Urban	Rural	*p*-value^#^	Urban	Rural	*p*-value^#^
	Mean ± SE	Mean ± SE		Mean ± SE	Mean ± SE		Mean ± SE	Mean ± SE	
	Mean ± SE	Mean ± SE		Mean ± SE	Mean ± SE		Mean ± SE	Mean ± SE	
Body Mass Index (kg/m^2^)	23.4 ± 0.37	21.9 ± 0.17	0.005*	25.8 ± 0.23	24.3 ± 0.16	<0.001*	24.5 ± 0.30	23.1 ± 0.15	0.002*
Systolic Blood Pressure (mmHg)	133.0 ± 0.94	131.3 ± 0.55	0.164	131.1 ± 1.72	128.3 ± 0.83	0.181	131.5 ± 0.95	130.0 ± 0.24	0.157
Diastolic Blood Pressure (mmHg)	83.0 ± 0.84	80.8 ± 0.32	0.037*	82.2 ± 1.19	80.9 ± 0.47	0.337	82.3 ± 0.81	80.9 ± 0.26	0.112
Fasting blood glucose(mmo/L)	5.9 ± 0.10	5.4 ± 0.06	0.001*	5.9 ± 0.08	5.5 ± 0.11	0.015*	5.9 ± 0.08	5.5 ± 0.09	0.003*
Total cholesterol (mmol/L)	5.5 ± 0.05	5.0 ± 0.09	0.002*	5.4 ± 0.05	5.4 ± 0.09	0.918	5.4 ± 0.05	5.2 ± 0.08	0.037*
Triglycerides (mmol/L)	1.9 ± 0.07	1.5 ± 0.07	0.002*	1.6 ± 0.04	1.4 ± 0.09	0.158	1.7 ± 0.05	1.4 ± 0.08	0.015*
High-density lipoprotein (mmol/L)	1.2 ± 0.01	1.3 ± 0.02	0.006*	1.1 ± 0.01	1.3 ± 0.01	<0.001*	1.1 ± 0.01	1.3 ± 0.01	<0.001*

^#^ *p*- value for differences between urban and rural values, *p-value <0.05 from Wald test

The prevalences of low physical activity, hypertension, diabetes and hypercholesterolemia were the highest in older age-groups and declined with decreasing age (data not shown in tables). Smoking and alcohol drinking were more common among younger participants (data not shown in tables).

The proportion of the population with more than 10% of chance of developing CHD during the next 10 years, based on the Framingham risk score, was significantly higher in the urban areas 41.6% (95% CI = 39.5–43.7) than in the rural areas 33.6% (95%CI = 29.8–37.3) (*p* = 0.006) (data not shown in tables). Age-standardized to the WHO world standard population, the estimates were 33.0% (31.7–34.4) and 27.0% (23.4–30.7) for urban and rural, respectively. In sub-groups by gender, the age-standardized 10% chance of developing CHD during the next 10 years was 33.0% (30.9–35.2) in urban females and 26.3% (24.4–28.3) in rural females. Corresponding figures for men were 51.9% (47.4–56.3) and 40.4% (36.0–44.8) in urban and rural areas, respectively.

## Discussion

Several of the NCDs risk factors were found to be highly prevalent, and the prevalence of metabolic risk factors, such as overweight and obesity, hypertension, hypercholesterolemia, hypertriglyceridemia and diabetes were highest among the urban residents, while the behavioral risk factors such as smoking, alcohol consumption and low fruit and vegetable intake were more commonly reported among rural people. Mean values of BMI, fasting blood glucose and lipids show more unfavorable values in urban than in rural areas. We report a significantly higher risk of developing CHD the next 10 years among urban than rural residents.

The present study had a high response rate in the urban (86%) and rural areas (83%), which reduces the likelihood of biased estimates due to selection out of the study. Institutionalized persons like nuns, monks and military were not invited to the study. The prevalences of NCD risk factors are not known for these groups of the population. If their health condition and health behavior differ from that of the included population, the reported prevalence estimates may be over or under estimates. It is, however, not likely that exclusion of institutionalized individuals has biased comparisons between urban and rural areas. We speculate that military personal may be more physical active and, having a lower blood pressure and, thus have a better risk profile leading to over estimation of our results. The nutrition, smoking or/and alcohol drinking habits may differ in various ways between the institutionalized persons and the general population, making it difficult to evaluate the inference of selection bias. Furthermore, if there is a difference in the population of institutionalized people in urban and rural areas, the reported urban-rural differences in prevalence of risk factors might be hampered. As more men than women are institutionalize (for instance- few female nuns, only 8404 registered religious females out of 3.8 million females (0.002% of females in Yangon Region) [8] and few female military personal), exclusion of institutionalized people may have affected estimates more among men than women. To be able to control for statistical bias, we accounted for complex sampling design effect and the sample weights in the analysis [30]. The older mean-age of the urban subjects than the rural subjects in the present study have not contributed significantly to the urban-rural differences in our results as the age-standardized estimates were reported. Further strengths of the study are that we have used an international accepted STEPs protocol and that fasting blood samples were analyzed at the same national reference laboratory for both the urban and the rural study, and that the data collection was conducted at the same time of the year in both areas. Self-reported measures for the behavioral risk factors are prone to recall bias, especially alcohol consumption. Homemade or local made alcoholic beverages contain unknown level of alcohol, and might, thus, give biased result in the calculation of units of alcohol consumed. However, we believe that information problems are equally distributed in urban and rural areas.

There is a potential for introducing bias during the interviewing of participants. In order to reduce the problem, we have carefully selected interviewers with a medical background (MDs), and they have received proper training by the principal investigator. and they collected data from approximately the same number of participants. We used a structured questionnaire and show-cards for questions on tobacco use, alcohol drinking, physical activity and fruit and vegetable intake, which may have further reduced potential interviewer bias in the present study. Another potential source of information problems is that the Framingham risk score has not been validated in Myanmar, only in other Asian populations. However, it is unlikely that the finding of an urban-rural difference is biased, but care should be taken in judging the exact estimates.

The daily smoking rate in the urban area is similar to that in the urban Cambodia (18.0%) [31], but lower than in the urban areas of Thailand (36.0%) [32] and India (22.6%) [33]. The smoking prevalence in rural Yangon is lower than in rural area of Thailand (33%) [32], however, similar to the prevalence seem in the rural of India (24.3%) [33] and Cambodia (28.2%) [31]. Similar to other figures from the region [34], daily smoking prevalence in males outnumber females, more than ten times in urban area and about three and a half time in rural area. The overall prevalence of smokeless tobacco (28.0% for urban areas and 32.5% for rural areas) is comparative to the 2009 nationwide figure (30.0%) [14, 15]. The results indicate that in both urban and rural areas, tobacco consumption among males stands as a public health problem in Myanmar, although more than 90% of smokers consumed less than ten tobacco units (cigarettes or cheroots) daily Tobacco use is a socio-culture entertaining habit in this society [35]. Poor education and economic status in the rural areas may enhance the use of tobacco. We recommend implementation and enforcement of law on tobacco control in order to reduce tobacco consumption, and to keep and even reduce the low figures among women.

The prevalence of current alcohol drinking from the 2009 national report (13.0%) [14] is comparable with our estimates in urban areas (13.9%) but is lower than the present rural prevalence (19.9%). Among males, the current alcohol drinking rate (28.4%) is comparable to urban Kerala, India (27%) [33]. Similarly to reports from Cambodia [31], few were heavy drinkers. It seems that heavy drinking is yet not a problem in the Yangon region or in Myanmar on average and the preventive measure should enforce to keep the low figures. There might be regions with high prevalence of alcohol drinking, and other unhealthy behavior that could be identified in regional STEPS survey.

The majority of the rural residents have very low income and poor education level. Moreover, the socio-culture custom, misconception about health behavior, for instance - that smokeless tobacco is less harmful than smoking [35], among this population could be a predisposing factor for high behavioral risk factors. This could be one of the explanations of higher prevalence of tobacco consumption and alcohol drinking among rural than urban people.

The large majority of the study population consumed inadequate amount of fruit and vegetables. The finding is consistent with the regional and global pattern [36, 37]. However, Myanmar is an agricultural country and should consider how to distribute and promote fruit and vegetable and its consumption. The nature of production, consumption and conception of fruit and vegetable in Myanmar could also be further studied, in order to facilitate an increased intake among its population.

Few participants reported low level of physical activity with no urban-rural difference. Our results are similar to the national figures from 2009 [14], and studies in India [33], Cambodia [31] and other low income countries [38, 39]. Physical activity is influenced by the physical and social environment such as urbanization, industrialization and economic development, social norms and motorized transport [38]. In accordance with studies from other developing countries [40], moderate to high physical activities among urban participants may reflect the daily work life and poor transportation systems in the city, as the present study revealed that physical exercise generally came from the occupational and walking or bicycling to the work place. The ongoing socio-economic development in Myanmar may reverse the current level of physical activity in urban areas unless the leisure time activity is increased accordingly. The trends in physical activity should be carefully monitored and interventions to promote physical activity should be addressed at a national level, especially for urban residents.

Similarly to other studies [31, 33], we found that the estimates of metabolic risk factors are generally worse for urban population than rural population. The results revealed a significant difference of overweight and obesity between urban-rural locations, also in sub-group analysis by gender. Compared with the much lower 2009 national figures (14% in male and 22% in female) [14], it could be that it reflects an increasing trend in overweight, which is consistent with the global trends. The global mean BMI has gradually increased and people becomes 1.5 kg heavier in every 10 years [41]. Despite no difference in physical activity between urban-rural locations, overindulgence in high calorie diet and indoor sedentary leisure activities may contribute the higher risk of overweight & obesity among urban dwellers.

We report an alarming and clearly higher prevalence of hypertension (40-41%) than global estimates (22%) and figures from SEAR region (24–25%) in 2014 [1]. As compared with national data from 2009 (30%) [14, 15], the prevalence of hypertension is higher in both urban and rural Yangon. Studies have reported that salt-intake in South-East Asian countries is high [42], and because salt and mono-sodium glutamate (MSG) intake are associated with high blood pressure [43–45], they could be a contributor to high prevalence of hypertension in Myanmar. The salt intake in Myanmar has not yet been estimated, but there are traditions for eating salty food, and salt has been used for preservation of food [46]. Risk factors of hypertension should be further studied in order to identifying preventive strategies of hypertension reduction.

The urban diabetes prevalence of 17.2% was higher than the global estimate of 2014 (7.9–9%) and regional SEAR figures (10.0%) [47]. Our rural diabetes prevalence estimate of 9.2% was close to global and regional estimates [47]. Studies from Asia have showed an increasing trend in diabetes prevalence, particularly in urban areas [48]. Urbanization with rapid changes in lifestyle in Asia could be predisposing factors for this increasing trend. It has also been suggested that Asian people has (epi)genetic predisposition for poorer threshold for the environmental risk factors and thus increased risk of diabetes [48]. The present study demonstrate high prevalence of overweigh and obesity, which could contribute to the present high diabetes values [1].

We report higher level of total cholesterol than the worldwide age-standardized mean total cholesterol level (4.64 mmol/L in males and 4.76 mmol/L in females) [49]. The prevalence of hypercholesterolemia has in several studies shown to be higher in urban than in rural areas: Cambodia [31], India [33] and Thailand [50], while in the present study differences were only significantly for males. Serum cholesterol is an etiological factor behind atherosclerotic cardiovascular diseases [28], and due to the finding of high prevalence of hypercholesterolemia in the present study, new studies should address associated factors, including type of oil used for preparing food.

Seventy percent of the 51 million Myanmar population lives in rural areas [8]. Due to a rapid urbanization rate (1.6%) [7], the proportion of people living in urban areas will soon increase considerably. The rural residents have more unhealthy behavioral risk factors for NCDs than their urban counterpart, but their metabolic measures are better than those living in urban areas. We are not able to explain why people of the rural area have better metabolic measures than urban residents, when they have worse behavioral factors. It could be due to unmeasured protective factors, unmeasured risk factors, or differences in foods within food categories. Unmeasured differences in types of oil used for food preparation between urban and rural areas could also be an explanation. Thus, it is not easy to predict the c development in short term, but for the long term it is likely that Myanmar will face an epidemic of NCDs. This prophecy is supported by our findings of a high 10 years risk of CHD in both urban (41.6%) and rural (33.6%) areas, and from findings in other studies that urban exposure increases the risk of CHD [51, 52]. Unless contextual integrated NCD control program is implemented, an increasing trend of NCD and their risk factors will continue in both urban and rural areas of Myanmar. Our prediction of higher CHD risk among urban than rural residents is in line with a systematic review in SEAR [53] and worldwide [54], and was consistent with a previous study from Yangon region [55]. Despite lower prevalence of smoking in urban than in rural areas, the significantly higher mean total cholesterol, diabetes prevalence, and mean age, and lower mean high-density lipoprotein level in urban area have contributed to the present higher 10 years risk of CHDs in urban area as compared to rural area. Gender differences were found in the prevalence of overweight and obesity, being higher in females than in males, while males had higher smoking, smokeless tobacco consumption and alcohol drinking prevalences than females. These results are in line with the findings in the other countries of South-East-Asia region [2].

The urban data in the present study was collected in one of the most developed urban areas of Myanmar, and might therefore not be generalizable to all urban areas. The rural data may differ from rural areas situated far from big cities. Thus, care should also be taken in generalizing findings to other urban and rural areas of Myanmar, especially the prevalence estimates. We did not include semi-urban residents in our study, and suggest inclusion of this group in upcoming studies to get a broader picture of the health of Myanmar populations.

## Conclusion

The prevalences of the majority of NCDs risk factors were high in both urban and rural areas of Yangon, Myanmar. Metabolic risk factors were more common among the urban residents whereas the behavioral risk factors were higher among rural people. The 10-year risk of CHD was significantly higher in the urban than in rural areas. Preventive measures are needed to avoid an increase in the risk of NCDs in Myanmar.

## Abbreviations

BMI: Body mass index
CHD: Coronary heart disease
DBP: Diastolic blood pressure
MET: Metabolic equivalent of tasks
NCD: Non-communicable disease
SBP: Systolic blood pressure
SEAR: South-East Asian Region
STEPs: STEPwise approach to non-communicable disease risk factor surveillance
